# Safety and efficacy of co-administration of CD19 and CD22 CAR-T cells in children with B-ALL relapse after CD19 CAR-T therapy

**DOI:** 10.1186/s12967-023-04019-4

**Published:** 2023-03-22

**Authors:** Wenjie Li, Lixia Ding, Wenhua Shi, Xinyu Wan, Xiaomin Yang, Jing Yang, Tianyi Wang, Lili Song, Xiang Wang, Yani Ma, Chengjuan Luo, Jingyan Tang, Longjun Gu, Jing Chen, Jun Lu, Yanjing Tang, Benshang Li

**Affiliations:** 1 Department of Hematology/Oncology, Key Laboratory of Pediatric Hematology & Oncology Ministry of Health, Shanghai Children’s Medical Center, School of Medicine, Shanghai Jiao Tong University, Shanghai, China; 2 Department of Hematology/Oncology, Children’s Hospital of Soochow University, Suzhou, Jiangsu China

**Keywords:** B-ALL, Second CAR-T, Co-administration, CD19/CD22 CAR-T

## Abstract

**Background:**

CD19-targeted chimeric antigen receptor T-cell (CAR-T) therapy has shown remarkable efficacy in treating relapsed or refractory pediatric B-lineage acute lymphoblastic leukemia (B-ALL). However, poor results are obtained when the same product is reused in patients who relapse after CAR-T. Therefore, there is a need to explore the safety and efficacy of co-administration of CD19- and CD22-targeted CAR-T as a salvage second CAR-T therapy (CART2) in B-ALL patients who relapse after their first CD19 CAR-T treatment (CART1).

**Methods:**

In this study, we recruited five patients who relapsed after CD19-targeted CAR-T. CD19- and CD22-CAR lentivirus-transfected T cells were cultured separately and mixed before infusion in an approximate ratio of 1:1. The total dose range of CD19 and CD22 CAR-T was 4.3 × 10^6^–1.5 × 10^7^/kg. Throughout the trial, we evaluated the patients’ clinical responses, side effects, and the expansion and persistence of CAR-T cells.

**Results:**

After CART2, all five patients had minimal residual disease (MRD)-negative complete remission (CR). The 6- and 12-month overall survival (OS) rates were 100%. The median follow-up time was 26.3 months. Three of the five patients bridged to consolidated allogeneic hematopoietic stem cell transplantation (allo-HSCT) after CART2 and remained in MRD-negative CR at the cut-off time. In patient No. 3 (pt03), CAR-T cells were still detected in the peripheral blood (PB) at 347 days post-CART2. Cytokine release syndrome (CRS) only occurred with a grade of ≤ 2, and no patients experienced symptoms of neurologic toxicity during CART2.

**Conclusions:**

Mixed infusion of CD19- and CD22-targeted CAR-T cells is a safe and effective regimen for children with B-ALL who relapse after prior CD19-targeted CAR-T therapy. Salvage CART2 provides an opportunity for bridging to transplantation and long-term survival.

*Trial registration*: Chinese Clinical Trial Registry, ChiCTR2000032211. Retrospectively registered: April 23, 2020.

**Supplementary Information:**

The online version contains supplementary material available at 10.1186/s12967-023-04019-4.

## Background

CD19-targeted chimeric antigen receptor T-cell (CAR-T) therapy induces a high rate of complete remission (CR) in children with relapsed or refractory B-lineage acute lymphoblastic leukemia (B-ALL) [1, 2]. However, CAR-T therapy does not yield a durable response, with relapse occurring in approximately 30–60% of patients [2–5]. Patients who relapse after a first round of CAR-T therapy (CART1) can be re-treated with the same CAR-T cells; however, the CR rate is reportedly as low as ≤ 40% [6–8]. Given the high relapse rate and the poor prognosis of B-ALL progression after CART1, there is an urgent need to find new strategies to treat relapse after CAR-T and to achieve long-term remission.

When administering a second round of CAR-T therapy after relapse (CART2), using CAR-T cells with different structures and targeting than in CART1 may lead to new responses. Most B-ALL cells express CD22, an alternative CAR-T therapy target [9]. Previous clinical trials suggest that immunotherapy targeting both CD19 and CD22 antigens may reduce antigen loss based on the evidence of CD19 antigen loss or downregulation following CD19-targeted immunotherapy and evidence that decreased CD22 expression contributes to relapse after CAR-T therapy [10–12]. Simultaneously, antigenic heterogeneity may enable a small fraction of tumor cells with dim/negative antigen expression to evade hits by a single targeted CAR-T. Dual-targeted CAR-T cells kill both single- and double-antigen-positive tumor cells, thereby preventing tumor escape allowed by antigenic heterogeneity [10, 13].

Dual-antigen models mainly include (1) pooled CAR-T in which two separately targeted CAR-T cells are co-administered, (2) bispecific CAR-T in which two different single-chain fragment variables (scFv) are connected in a single CAR vector, or (3) bicistronic CAR-T, in which two individual CARs can exist in the same T cell [14, 15]. Our institution has achieved a 99% minimal residual disease (MRD)-negative CR rate with co-administration of CD19 and CD22 CAR-T among children with B-ALL, seemingly better than results with single-targeted CD19 or CD22 and other dual-targeted structures [16–21].

Here we conducted a clinical trial to explore the safety and efficacy of CD19 and CD22 CAR-T co-administration as CART2 in B-ALL patients who relapsed after CD19 CART1. This article presents patient baseline data, treatment characteristics, and side effects after CART2. Furthermore, we monitored CAR-T expansion and persistence to evaluate treatment effects. In patients who relapsed after CART1, CART2 therapy brought a re-remission, thus providing an opportunity for bridging transplantation and long-term survival.

## Methods

### Study design

This single-arm, multicenter, phase I study included five patients ≤ 18 years old who relapsed after CD19 CAR-T and received co-infusion of CD19- and CD22-targeted CAR-T cells between July 2018 and August 2022. The study was conducted at Shanghai Children’s Medical Center, School of Medicine, Shanghai Jiao Tong University, and Children’s Hospital of Soochow University. It was approved by the institutional review board of both institutions and registered in the Chinese Clinical Trial Registry (ChiCTR2000032211). Detailed inclusion and exclusion criteria are provided in the supplementary (Additional file 1). For each patient, written informed consent was signed by the parents, guardians, or patients, and the study was conducted in accordance with the Declaration of Helsinki. The cut-off time for follow-up was August 31, 2022.

### Generation of CAR-T cells

CAR-T infusion was considered as day 0. On day − 7, the appropriate amount of whole blood was collected from patients through peripheral veins, according to weight and lymphocyte count. T cells were isolated from peripheral blood (PB) by density gradient centrifugation using Ficoll-Paque^™^ PREMIUM (Cytiva, Sweden, AB) and anti-CD3 Dynabeads^™^ (Invitrogen, Lithuania). On day − 5, after a 2-day activation with anti-CD3/CD28 Dynabeads^™^ (Gibco, Lithuania), T cells were separately transduced with CD19- and CD22-targeted second-generation CAR lentivirus, both with a 4-1BB costimulatory and CD3ζ signaling domain. After transduction, CD19 and CD22 CAR-T cells were expanded separately in vitro with TexMACS GMP Medium (Mitenyi, Germany) supplemented with IL-7 and IL-15 (Mitenyi, Germany). CAR-T cell numbers and viability were calculated daily using Trypan blue (Gibco, USA). On day − 3, the transduction efficiency of CD19 and CD22 CAR was detected by flow cytometry with F(ab)’2-Goat anti-Mouse IgG (H + L) Cross-Adsorbed Antibody, Biotin (Invitrogen, USA) and Streptavidin, R-Phycoerythrin Conjugate (Invitrogen, USA). After approximately 7 days of culture, the numbers of CD19 and CD22 CAR-T cells were calculated separately, mixed, and injected into patients in a single injection at a ratio of about 1:1. The availability of manufactured CAR-T cells was defined as the total dose range of CD19 and CD22 CAR-T of 1 × 10^6^/kg to 2 × 10^7^/kg with transduction efficiency ≥ 20% and viability rate > 90%.

### Lymphodepletion (LD) chemotherapy pretreatment

All patients received pre-CART1 LD chemotherapy, which included 40 mg/m^2^ fludarabine (Flu) on days − 4 to − 2, and 500 mg/m^2^ cyclophosphamide (Cy) on days − 4 to − 3 (with day 0 being the day of CAR-T infusion). LD before CART2 comprised a higher dose of Flu (50 mg/m^2^) for three days and 500 mg/m^2^ Cy for two days. All patients received fresh CAR-T cells during both CAR-T treatments.

### Tumor burden assessment

Bone marrow (BM) aspirates were taken for cytomorphological and MRD analysis to assess tumor burden at the time of enrollment, day 0 (after lymphodepletion chemotherapy and before CAR-T infusion), day 7 − 14 (at any time during the period), day 28, and 3, 6, 12 months post CAR-T administration. May-Grunwald Giemsa stained bone marrow aspirate slides were evaluated with an Olympus light microscope by a pathologist. The number of blasts in 500 nucleated cells was calculated using a 100-fold oil immersion lens. The morphological assessment was categorized as M1 (< 5% blasts), M2 (5 − 25% blasts), and M3 (> 25% blasts) bone marrow. Flow cytometry (FCM) assessment of MRD was performed according to the guidelines of Shanghai Children’s Medical Center. BM aspirates were collected into heparin lithium tubes (BD, NJ, USA), and cells were stained with an antibody panel composed of CD10-PECy7/ CD34-PerCP/ CD22-APC/ CD19-APCH7/ CD20-V450/ CD45-V500 (BD, San Jose, CA, USA). Data were acquired on the 8-color BD FACS Canto II FCM (San Jose, CA, USA) and analyzed by BD FACSDiva software and Flowjo version 10. MRD was calculated as the percentage of tumor cells to mononuclear cells.

In patients with central nervous system (CNS) involvement, CSF was collected by lumbar puncture and analyzed by FCM to detect blasts at the same time points as BM aspirations. For non-CNS extramedullary diseases, e.g., in the lymph nodes or testis, tumor size, texture changes, and the presence of leukemia cells are measured by observation, ultrasound, or biopsy to evaluate the efficacy of CAR-T therapy.

### Efficacy evaluation

CR was defined as no circulating blasts and extramedullary disease, including negative CSF and < 5% BM blasts with full hematological recovery (platelets > 100,000/µL and absolute neutrophil count > 1000/µL) [16, 22]. MRD-negative CR was defined as < 0.01% leukemia cells detected in BM by FCM. Relapse was defined as the reappearance of blasts in BM, PB, or an extramedullary site after achieving MRD-negative CR. The objective response rate (ORR) was the incidence of MRD-negative CR after CART2 infusion. Event-free survival (EFS) was defined as the time from CART2 infusion to events, including relapse, bridging to transplantation, or death [2]. Patients were censored at allo-HSCT and lost to follow-up, although possibly without disease progression. Overall survival (OS) was defined as the time from CART2 infusion to the last follow-up or death.

### Safety evaluation

The BD^™^ Cytometric Bead Array (CBA) Human Th1/Th2/Th17 Cytokine Kit (San Jose, CA, USA) was used to analyze serum concentrations of released cytokines, including interleukin (IL)-2, IL-4, IL-6, IL-10, IL-17A, tumor necrosis factor α (TNF-α), and interferon γ (IFN-γ). Cytokine release syndrome (CRS) was graded according to the American Society for Transplantation and Cellular Therapy (ASTCT) grading system [23]. Neurologic toxicity was graded in accordance with the Common Terminology Criteria for Adverse Events (CTCAE) 4.03 [24].

### CAR-T expansion and persistence

5 ml PB was collected on day 2, 7, 28 and 2, 3, 4, 5, 6, 9, and 12 months after CAR-T infusion to detect CAR-T count and copy number, which were used to assess expansion and persistence of CAR-T cells. FCM was used to evaluate the ratio of CAR-T cells to CD3^+^CD45^+^ T cells and the absolute number of CAR-T cells per μL of PB, namely, CAR-T count. Quantitative polymerase chain reaction (qPCR) for CD19 CAR, CD22 CAR, and total CAR-T gene copies was conducted on genomic DNA extracted from PB (TIANGEN, BEJ) using Hieff^™^ SYBR^®^ GREEN Master Mix (Yeasen, SHH). CAR copies per μg DNA were normalized by the single-copy gene CDKN1a.

### Statistical analysis

Descriptive statistics were used to characterize patient baseline characteristics. EFS and OS were estimated using Kaplan–Meier curves. Fold expansion and the absolute value of IL-6 after CART1 and CART2 were analyzed by Mann–Whitney U test. Data were processed using GraphPad Prism 9. A two-tailed *P* value of < 0.05 was considered to indicate statistical significance.

## Results

### Patient baseline and CART1 therapy characteristics

The baseline characteristics of the five included patients are presented in Additional file 2: Table 1. The median age at diagnosis was 3.62 years (range, 1.39–11.87 years), and the median age at CART1 was 7.36 years (range, 5.61–12.38 years). Three patients had cytogenetic abnormalities, among whom one patient with BCR-ABL1 fusion. Before CART1, four patients had a high disease burden (MRD ≥ 5% or extramedullary disease), and only one patient had a low disease burden (MRD < 5%). No patients had received CAR-T therapy before CART1. All patients received LD pretreatment (Flu 40 mg/m^2^ and Cy 500 mg/m^2^). The median dose of CART1 was 1 × 10^7^/kg (range, 8 × 10^6^–1.2 × 10^7^/kg). All patients exhibited objective responses to CART1 but then experienced CD19-positive relapse (Additional file 2: Table 1).

### Disease status and treatment characteristics at CART2

**Fig. 1:**
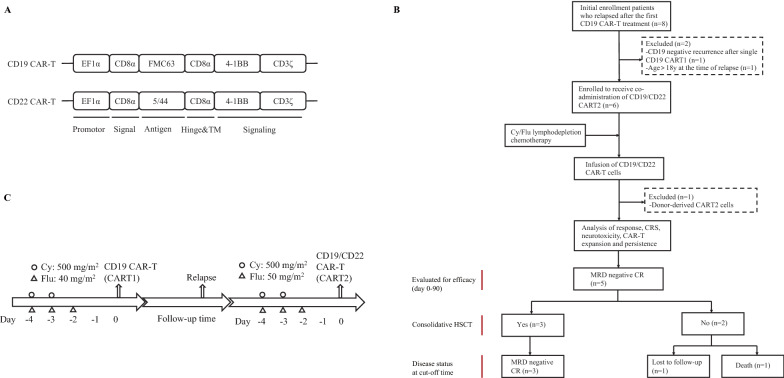
Schematic diagram of CD19/CD22 CAR-T structure, clinical trial enrollment, and design. **A** Schematic representation of the transgenes used for the two CAR-T constructs targeting CD19 and CD22. **B** Flow chart of patient recruitment and treatment evaluation. All patients achieved MRD-negative CR after CART2. Three patients who bridged to HSCT after CART2 were still in MRD-negative CR at the cut-off time. Of the two patients who did not receive HSCT after CART2, one died and the other was lost to follow-up. **C** A simplified diagram of the lymphodepletion chemotherapy conditioning regimen administered before each of the two CAR-T treatments

The median age at CART2 infusion was 8.36 years (range, 6.96–15.1 years). The median time from CART1 infusion to relapse was 11.87 months (range, 4.07–32.87 months), and the median time between CART1 and CART2 was 15.93 months (range, 11.9–33.13 months). At the time of CART1 failure, BM was the most common disease site, with three patients exhibiting isolated bone marrow relapse. Another two patients relapsed with isolated extramedullary disease: testis and lymph node, respectively (Additional file 3: Table 2). All patients underwent LD chemotherapy before CART2 (Flu 50 mg/m^2^ and Cy 500 mg/m^2^). The median CART2 dose was 1.1 × 10^7^/kg (range, 4.3 × 10^6^–1.5 × 10^7^/kg), among which the median dose of CD19 CAR-T is 5.54 × 10^6^/kg (range, 2.21 × 10^6^–6 × 10^6^/kg), and the median dose of CD22 CAR-T is 5.29 × 10^6^/kg (range, 2.09 × 10^6^–9 × 10^6^/kg). The median ratio of CAR-T doses of CD19 to CD22 was about 1:1 (range, 1:0.9–1:1.5). For CART2, the median average transduction efficiency was 44.85% (range, 25%–64.55%). Three patients received higher doses for CART2 than CART1; one was treated with the same dose in both CAR-T treatments, and one received a lower dose for CART2 (Additional file 3: Table 2). Figure 1A–C shows the CD19/CD22 CAR-T structure, clinical trial enrollment, and design schematic diagram.

### CRS and neurologic toxicity after CART2

This study observed no cases of ≥ grade 3 CRS or CAR-T-related death after CART2. Five patients developed mild CRS (grade 1 in three patients and grade 2 in two patients) in CART2 (Additional file 3: Table 2). All patients developed a fever (≥ 38℃) within 1 day after CART2, of whom two patients exhibited low blood pressure but without requiring vasopressor treatment. Tocilizumab was administered to two patients with grade 2 CRS, and none received dexamethasone. CRS-related symptoms were transient and reversible in all patients. No patients experienced symptoms of neurologic toxicity, such as headache, cognitive changes, convulsions, etc. (Additional file 3: Table 2).

**Fig. 2:**
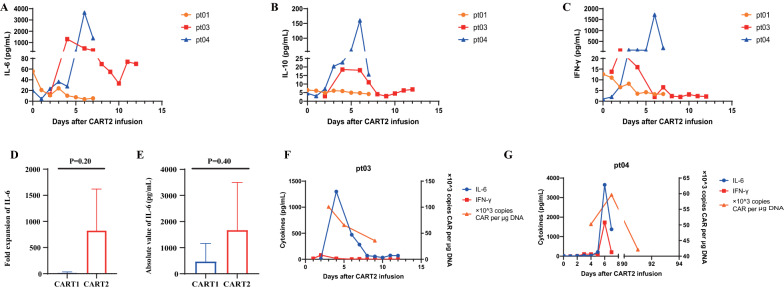
After CAR-T infusion, patients were monitored for cytokines related to CRS and CAR-T cell expansion. **A**–**C** The levels of serum cytokines IL-6, IL-10, and IFN-γ after CART2 infusion. **D**–**E** Fold increase and the absolute value of IL-6 in CART1 and CART2 were analyzed by Mann − Whitney U test. The expansion of IL-6 was much higher after CART2 than CART1, although the *P* value did not reach statistical significance (*P* = 0.2; *P* = 0.4). **F**–**G** The trends of change in IL-6, IFN-γ, and CAR transgene copies in PB were similar after CART2

Cytokines, such as IL-6, IL-10, and IFN-γ, derived from CAR-T cells and bystander monocytes are essential components leading to CRS production (Fig. 2A–C), among which IL-6 is the central biomarker [25]. The increase of IL-6 level after CAR-T infusion was considered the beginning of CRS, and the return of IL-6 to the baseline level was considered the end of CRS [26]. CRS onset was usually within 6 days after CART2 infusion, and CRS lasted for 7–14 days (Fig. 2A–C). The peak serum cytokine levels of IL-2, IL-4, IL-6, IL-10, IL-17A, IFN-γ, and TNF-α after CART2 are presented in Additional file 4: Table S1. The fold expansion of IL-6 refers to the ratio of its highest value after CAR-T infusion to the pre-CART baseline level. The median fold change of IL-6 was observed to be more elevated after CART2 than after CART1 (7.46 vs. 849.7, *P* = 0.2), as was the median peak absolute number of IL-6 (100.4 vs. 1300 pg/mL, *P* = 0.4) (Fig. 2D–E). Serum levels of IL-6 and IFN-γ were the representative indicators of CAR-T amplification ability [27, 28], which were consistent with the changing trend of CAR-T copy number after CART2 (Fig. 2F–G).

### CAR-T cell response characteristics after CART2

**Fig. 3:**
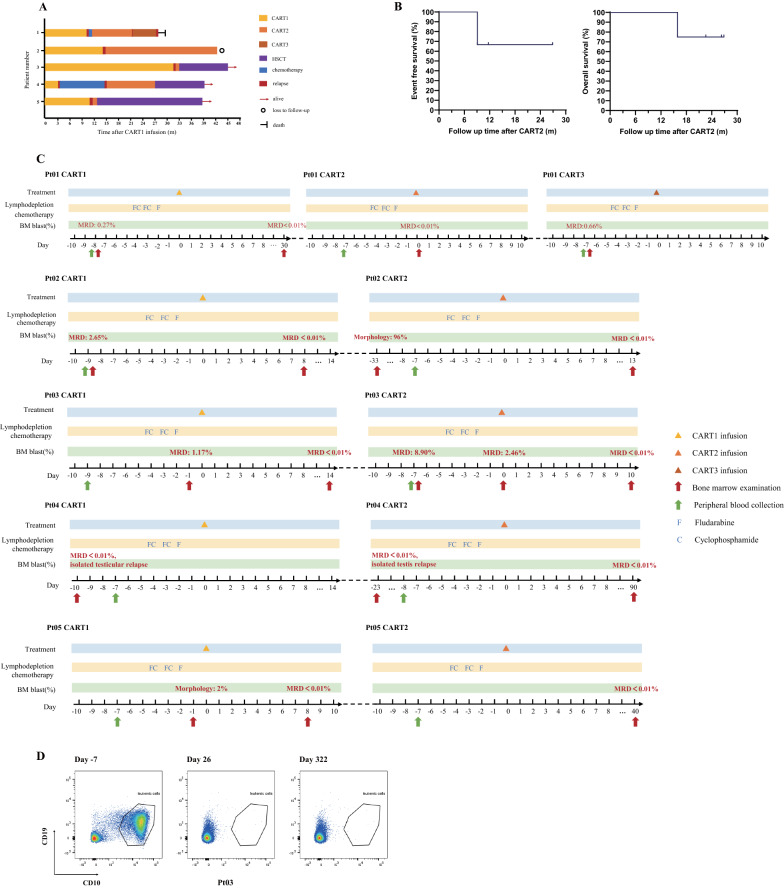
Treatment process and outcomes. **A** The whole treatment process for five patients from CART1 infusion to the cut-off time. Each bar's color and length represent the status and duration of the response, respectively. Pt04 received salvage transplantation after CART2. **B** Kaplan–Meier curves of event-free survival (EFS) and overall survival (OS) probabilities in CART2. **C** The detailed treatment process of 5 patients, including the time and result of BM aspiration, the scheme of lymphodepletion chemotherapy, and the time of CAR-T infusion. **D** In pt03, the proportion of leukemia cells changed from MRD 8.9% on day − 7 to MRD < 0.01% on day 26 and remained at MRD < 0.01% on day 322 after CART2 (post HSCT)

Figure 3A shows the treatment course of five patients from CART1 infusion to the cut-off time. The median follow-up time was 26.3 months. The ORR was 100%. All patients achieved MRD-negative CR assessed by flow cytometry. After CART2, EFS was 100% at 6 months and 80% at 12 months. The 6-month and 12-month OS were 100% (Fig. 3B). The time interval with available data from CART2 reinfusion to MRD-negative CR was 0–3 months. The detailed treatment process of 5 patients is shown in Fig. 3C, including the time and result of BM aspiration, the scheme of LD chemotherapy, and the time of CAR-T infusion. In pt01, B-ALL recurred 9 months after CART2 infusion, and the patient received a third CAR-T treatment because parents refused the follow-up transplantation. Unfortunately, pt01 relapsed after salvage therapy, and parents ultimately abandoned the treatment. In pt03, the proportion of leukemia cells changed from MRD 8.9% on day − 7 to MRD < 0.01% on day 26 and remained at MRD < 0.01% on day 322 after CART2 (post HSCT). Changes in MRD before and after CART2 reflect the killing ability of CD19-/CD22- CAR-T cells (Fig. 3D).

### Expansion and persistence of CAR-T cells after CART2

**Fig. 4:**
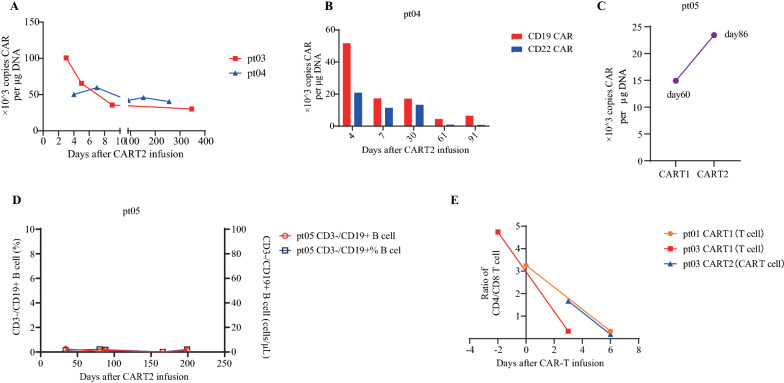
Expansion and persistence of CAR-T cells after CART2. **A** CAR-T copy number in PB detected by qPCR. **B** Transgenic copy number of each component of the CART2 treatment: CD19 and CD22 CAR-T cells in pt04. **C** In pt05, the vector copy number at 89 days after CART2 was higher than at 60 days after CART1. **D** Pt05 continued to be in a state of B-cell aplasia after CART2, which was reflected by the proportion of CD19^+^ B cells (%) and the absolute number of B cells (cells/μL). **E** Changes in the ratio of CD4/CD8 T or CAR-T cells around CAR-T infusion

Two patients with available data had significant CAR-T cell expansion based on the real-time qPCR detection of CART2 transgene copies in PB (peak values of CAR-T copy number were 100.65 × 10^3^ CAR copies/µg and 59.63 × 10^3^ CAR copies/µg in pt03 and pt04, respectively). CAR-T copy number typically peaked within 1 week after CAR-T infusion and then gradually declined. In pt03, CAR-T cells could still be detected in PB at 347 days after CART2 reinfusion (Fig. 4A). In pt04, CD19 CAR-T had earlier and more robust expansion with longer duration than CD22 CAR-T (Fig. 4B), which was consistent with previous findings [16]. In pt05, 23.45 × 10^3^ CAR copies/µg DNA were detected in PB at 89 days after CART2 infusion, which was dramatically higher than the 14.93 × 10^3^ CAR copies/µg DNA detected at 60 days after CART1 infusion (Fig. 4C).

The study by Wang et al. showed that persistent B-cell aplasia (BCA) at 6 months or beyond after CAR-T infusion was related to favorable outcome [16]. BCA was defined as a < 1% proportion of CD3^−^CD19^+^ B cells or an absolute B-cell number of ≤ 200/µL among lymphoblasts in BM or PB. The median duration of BCA in CART1 was 6.03 months (range 3.17–9.77 months). The median duration of BCA in CART2 was 11.57 months (range 8.53–17.5 months), which was longer than that in CART1 (Additional file 3: Table 2; Fig. 4D).

The initial infusion of CAR-T products and PB included more CD4^+^ T cells than CD8^+^ T cells; however, the CD4/CD8 ratio gradually decreased and inverted within 6 days after CAR-T reinfusion. This observation suggested that the more significant expansion of CD8^+^ T or CAR-T cells, relative to CD4^+^ T cells, reflected their central role in B-ALL cell elimination (Fig. 4E). Additionally, the CD4/CD8 ratio may be an indicator of whether a patient responds to CAR-T treatment. If the CD4/CD8 ratio remains unchanged before and after infusion, it might be a nonresponse to CAR-T [29]. In our study, all patients with available data had a reduced CD4/CD8 ratio after infusion and were responsive to CAR-T (Fig. 4E).

## Discussion

Although CD19 CAR-T cells have achieved outstanding therapeutic effects, B-ALL relapse after CAR-T therapy remains a thorny problem to be solved. Retreatment with the same CAR-T cells is likely to produce a clinical response but may not achieve durable remission [8]. Here we analyzed the safety and efficacy of co-administering separate CD19 and CD22 CAR-T cells for the treatment of relapse after CD19 CART1 in five patients. Our results demonstrated that CD19 and CD22 CAR-T cells co-administered as CART2 could significantly expand and exhibit good persistence based on ensuring safety. Meanwhile, allo-HSCT could be administered as consolidation therapy to prolong survival further.

No patient experienced CRS of over grade 2, and the CRS clinical symptoms were temporary and reversible, supporting the safety of CART2. IL-6 is one of the core cytokines during CAR-T therapy; it is a CRS biomarker and positively correlated with the expansion of CD19 CAR-T cells [30, 31]. We found that the multiplicative levels and absolute value of IL-6 were higher in CART2 than those in CART1, which may indicate that CART2 showed superior amplification compared to CART1. However, due to the limited data volume in our present study, more cases are needed to verify the correlation between IL-6 levels and CAR-T expansion.

Compared to similar clinical trials that have reused CAR-T cells to treat B-ALL relapse, our study seems to have achieved better treatment results [6–8]. Importantly, all patients in our study achieved MRD-negative CR after CART2. The 6-month and 12-month OS were 100%. The superior results of our trial may be attributed to higher doses of CART2 and the combination of CD19/CD22 dual-target CAR-T cells [12].

Several trials have validated the correlation between higher CAR-T cell dose and patient prognosis. Stefanski and colleagues [32] showed that patients who received higher doses of CD19 CAR-T cells exhibited significantly better EFS and OS and did not develop additional toxicity from the treatment. This conclusion was confirmed by Gauthier et al. [6], who suggested that an increased CART2 dose promoted CAR-T cell proliferation and persistence, leading to better treatment outcomes and that a high CART2 dose was an independent factor contributing to a superior overall response rate. In this trial, the median CART2 dose was 1.1 × 10^7^/kg, whereas other similar studies have reported median CART2 doses of 1–3 × 10^6^/kg [6–8, 21]. Among 5 patients, pt03 and pt05 received higher doses of CAR-T cells in CART2 (1.1–1.2 × 10^7^/kg) than in CART1, and both patients remained in MRD-negative CR at the data cut-off. In pt03, CAR-T cells were still detectable in the PB at day 347 after CART2 reinfusion. In pt05, a higher copy number of the CAR transgene was measured at day 89 after CART2, compared to day 60 after CART1. In conclusion, these two sets of data reflect the possibility that increasing CART2 dose may promote CAR-T persistence and amplification, resulting in higher response rates [6–8].

Co-administration of CD19 and CD22-targeted CAR-T cells, rather than single-targeted cells or sequential therapy, may reduce excessive pressure on a single target, thereby avoiding antigen reduction or loss. Tumor cells respond to the immune pressure of CAR-T by altering their expression of target antigens, such as by reducing antigen expression below the threshold required for CAR-T cell activation or losing detectable antigens, which can prevent the killing by CAR-T cells and even enable tumor recurrence [33]. In a prior study, among eight patients who relapsed after induction of remission with sequential CD19 and CD22 CAR-T therapy, decreased or complete loss of CD22 locus density developed in seven patients, and CD19 and CD22 negative relapse developed in one patient [10]. Among our five patients, none experienced CD19 or CD22 negative relapse after CART2.

At the same time, the expression levels of antigens on the surface of tumor cells are variable, and differential expression exists not only in different tumor cells within a single tumor but also in different individuals with the same type of tumor, which reflects tumor heterogeneity. Co-administration of CD19 and CD22 CAR-T cells can kill CD19^+^CD22^−^, CD19^−^CD22^+^, and CD19^+^CD22^+^ tumor cells, thereby avoiding tumor escape caused by heterogeneous expression of antigens [10, 34].

Considering the high relapse risk after CART2, allo-HSCT is recommended as consolidation therapy to prolong durable remission [7]. In our study, 3 patients were bridged to HSCT after CART2 and remained in MRD-negative CR at the cut-off time. Of the 2 patients who did not receive HSCT after CART2, one died, and the other was lost to follow-up. Although the number of patients included in our study was limited, the prognosis of the bridging HSCT group after CART2 was superior to that of the non-bridging HSCT group. Therefore, in combination with previous studies, we believe post-CART2 bridging transplantation deserves a positive recommendation [17].

One patient (pt01) in our study experienced another CD19 and CD22 positive relapse 9 months after CART2. There could be multiple reasons for the recurrence after CART2, including low tumor burden, inadequate CAR-T activity, and anti-murine CAR immunogenicity.

Dourthe et al. and Gardner et al. reported that low disease burden or antigen density was associated with antigen-positive relapse [35, 36]. After pretreatment with lymphodepletion, pt01 had a low tumor burden (MRD < 5%) on day 0. Lack of CD19 and CD22 antigens that stimulate T cell expansion may be involved in CAR-T cell disappearance and CD19^+^CD22^+^ relapse. In addition, compared with CD19 CAR-T, CD22 CAR-T has weaker expansion ability and shorter persistence, so the insufficient activity of CD22 CAR-T may not be able to inhibit the growth of tumor cells [16]. Current approaches to improve CAR-T cell activity and duration may include (1) regular replenishment of new CAR-T cells, (2) sorting and enrichment of naïve CAR-T cells, (3) development of novel scFv to improve CAR-T activity, etc. [37].

Previous research has indicated that the existence of anti-murine reactive T cells and antibodies targeting partial epitopes within a murine scFv is likely to impair CAR-T persistence and expansion, leading to poor outcomes for patients who proceed to CART2 using the same murine-derived CAR-T product as used in CART1 [6, 38]. Although Mueller et al. [39] reported that cellular and humoral responses to murine CD19 CAR-T did not impact therapeutic efficacy and safety in B-ALL, their study was based on CAR-naïve cohorts. Anti-murine CAR immune responses may have a more noticeable impact in CART2 than in CART1, as verified in five patients reported by Turtle et al. [40]. Compared to the first exposure, the immune system tends to mount a more vigorous and faster immune response to the second invasion of CAR-T by immunogenic scFv.

A combination of humanized CAR-T therapy may circumvent immunogenicity, thereby reducing humoral or cellular immune-mediated rejection. Myers et al. used humanized CD19 CAR-T to treat patients who relapsed after murine-derived CAR-T therapy and reported a 1-month ORR of 64% [41]. Similarly, An et al. observed a CR rate of 68% in their cohort [42]. Further investigation is needed to determine whether the recurrence in pt01 after CART2 was attributable to anti-murine CAR immunogenicity. Patients with antigen-positive relapse could be monitored for anti-CAR antibodies or reactive T cells before reinfusion of CAR-T cells.

## Conclusions

In conclusion, our findings suggest that co-administration of CD19- and CD22-targeted CAR-T cells may represent a new approach for treating patients who have relapsed after prior CD19 CAR-T therapy. An enhanced CAR-T dose and dual-targeted CAR-T may improve outcomes after CART2. Simultaneously, allo-HSCT, as a consolidation regimen following CART2, is likely to have potential benefits in terms of disease prognosis. Considering the influences of CAR-T cell persistence and viability, anti-CAR immunogenicity, and other factors on CART2, further exploration of other methods to optimize the efficacy of therapy is warranted.

## Supplementary Information

**Additional file 1:** Inclusion and exclusion criteria.

**Additional file 2: Table 1.** Patient characteristics and response to CART1. -: no follow-up. ^: CSF detected by flow cytometry indicated that immature cells account for 88.63% of nucleated cells. &: CSF detected by flow cytometry showed white blood cell count 25/mm^3^ and immature cells account for 84% of nucleated cells. *: The biopsy revealed infiltration of leukemia cells in the testis.

**Additional file 3: Table 2.** Disease status and treatment outcomes at CART2.-: no follow-up. *, MRD-negative CR was obtained after chemotherapy. CART2 was performed to prevent a recurrence. #, Pt01 relapsed after CART2 and received a third CAR-T salvage therapy. + : On the fourth day after CART2 reinfusion, pt04 began to develop testicular edema, along with redness and pain. After a week, the mass gradually shrank, and the tissue returned to normal size.

**Additional file 4: Table S1.** Peak values per cytokine in CART2 (pg/mL). The peak serum cytokine levels of IL-2, IL-4, IL-6, IL-10, IL-17A, IFN-γ and TNF-α in pt01, pt03 and pt04 after CART2.

## Data Availability

The datasets supporting the findings of this study are available from the corresponding author on reasonable request.

## Abbreviations

Allo-HSCT: Allogeneic hematopoietic stem cell transplantation
ASTCT: American Society for Transplantation and Cellular Therapy
B-ALL: B-lineage acute lymphoblastic leukemia
BCA: B-cell aplasia
BM: Bone marrow
CAR-T: Chimeric antigen receptor T cell
CBA: Cytometric bead array
CNS: Central nervous system
CR: Complete remission
CRS: Cytokine release syndrome
CSF: Cerebrospinal fluid
CTCAE: Common terminology criteria for adverse events
Cy: Cyclophosphamide
EFS: Event-free survival
Flu: Fludarabine
MRD: Minimal residual disease
ORR: Objective response rate
OS: Overall survival
PB: Peripheral blood
qPCR: Quantitative polymerase chain reaction
scFv: Single-chain fragment variables
