# Supplementary material for: Safety and efficacy of co-administration of CD19 and CD22 CAR-T cells in children with B-ALL relapse after CD19 CAR-T therapy
Source: J Transl Med. 2023 Mar 22;21:213. doi: 10.1186/s12967-023-04019-4 (PMC10031882; doi:10.1186/s12967-023-04019-4)
Supplement: Supplementary file 1 — Additional file 1: Inclusion and exclusion criteria. [file 12967_2023_4019_MOESM1_ESM.docx]

**Inclusion Criteria**

1. Patients with relapsed acute lymphoblastic leukemia (ALL) who did not achieve complete response (minimal residual disease (MRD) <0.01% or <5% blasts in bone marrow) after ≥2 courses of remission induction treatment or patients with refractory ALL with MRD >1% at the end of remission induction treatment and durable >0.1% after consolidation treatment;
2. Patients in whom prior CD19 CAR-T therapy failed;
3. Patients who relapsed at least 100 days after allogeneic hematopoietic cell transplantation (allo-HSCT), without active graft-versus-host disease (GVHD) and no longer taking immunosuppressive agents for at least 1 month prior to enrollment;
4. Patients with Philadelphia chromosome-positive ALL who are unable to tolerate tyrosine kinase inhibitor therapy or who experienced relapsed or refractory ALL after treatment with at least two distinct types of tyrosine kinase inhibitors;
5. Patients with relapsed or refractory ALL with high-risk genetic subtypes, e.g., hypodiploid (<44 chromosomes), TCF3–HLF-positive, or MEF2D-positive ALL;
6. Patients whose leukemic cells express fusion genes involving KMT2A or ZNF384;
7. Patients with isolated extramedullary relapse of ALL, including isolated testicular or central nervous system (CNS) relapse;

**Exclusion Criteria**

1. Age >18 years;
2. Karnofsky performance score <50 for patients ≥16 years or Lansky performance score <50 for those <16 years;
3. Active autoimmune disease or history of autoimmune disease involving the central nervous system;
4. Uncontrolled virus infection, including hepatitis B (HBV) or C (HCV) or human immunodeficiency virus (HIV);
5. Uncontrolled, active infection;
6. Active CNS dysfunction, e.g., uncontrolled seizure, paralysis, dementia, or cerebrovascular hemorrhage;
7. History of other types of malignant tumors;
8. Acute respiratory failure;
9. Acute heart failure or severe arrhythmia;
10. Serum creatinine/ blood urea nitrogen over 1.5 times than the upper limit of normal;
11. Disease progression too rapid to complete the clinical trial;
